# Handgrip Strength is Associated with Psychological Functioning, Mood and Sleep in Women over 65 Years

**DOI:** 10.3390/ijerph16050873

**Published:** 2019-03-10

**Authors:** José Alberto Laredo-Aguilera, Juan Manuel Carmona-Torres, Ana Isabel Cobo-Cuenca, Felipe García-Pinillos, Pedro Ángel Latorre-Román

**Affiliations:** 1 Department of Nursing, Universidad de Castilla-La Mancha, 45600 Talavera de la Reina, Spain; josealberto.laredo@uclm.es; 2 Department of Nursing, Universidad de Castilla-La Mancha, 45071 Toledo, Spain; anaisabel.cobo@uclm.es; 3 Department of Physical Education, Sport and Recreation, Universidad de La Frontera, Temuco 4813273, Chile; fegarpi@gmail.com; 4 Department of Corporal Expression, Universidad de Jaén, 23071 Jaén, Spain; platorre@ujaen.es

**Keywords:** hand strength, depression, sleep, moods, aged

## Abstract

Background: The predictive nature of handgrip strength (HGS) was analyzed, showing a direct association with the functional domains of health, cognitive and social levels, and some inverse association with depressive values. Aim: To analyze the relationship between HGS and the psychological functioning of older people, such as depression, mood and sleep. Method: A total of 38 women, participated in this study (age = 72.29 ± 5.21 year). As measurement instruments a hand dynamometer was used for HGS, Profile of Mood Status (POMS) 29 was used for mood, the geriatric depression scale was used for depression, and the Oviedo questionnaire was used for sleep. A cluster analysis was performed taking into account the performance in the HGS. Results: The group that obtained a high HGS result showed a better total score for vigor, depression, insomnia and sleep. Pearson correlation analysis showed significant correlations between HGS and vigor, depression, insomnia and sleep total score. Conclusion: HGS in women over 65 years was associated with psychological functioning and sleep quality.

## 1. Introduction

In today’s society, life expectancy is increasing exponentially compared to previous decades, with life expectancy for men now 80.1 years and for women 85.6 years, and a forecast for the year 2063 of 91.0 years in men and 94.3 years in women [1]. Adding low birth rates highlights the problem of an aging society, together with its associated factors, and it is estimated that this trend of an aging population will continue to increase [2].

In the aging process, there is a reduction of functional reserves, increased sensitivity to external aggressions, and reduced response mechanisms, causing fragility, disability, falls, sarcopenia, and the reduction of mass and muscular qualities directly related to the possibility of performing the basic and instrumental activities of daily life, which reduces the quality of life [3]. Aging also affects psychological function, perception of reality, and relationships with others and with oneself, conditioning the person’s cognitive way of reacting and affectively altering the feeling, acting, and thinking of the older people, in turn affecting their lives [4]. Good physical health and the absence of mental disorders are necessary but not sufficient conditions for positive mental health. To maintain psychological well-being, it is necessary to possess correct self-acceptance, positive relationships with others, a domain of the environment, autonomy, purpose in life, and personal growth, some of these being affected by the aging process [5].

As aging progresses, the sleep habits of older people change, including for example a reduction in the duration and effectiveness of sleep, increased latency and sleeplessness at night, and increased frequency of daytime sleep [6,7,8]. Obesity is another chronic disease that is currently a global problem due to its increased prevalence in recent years that also adds to the sleep disorders caused by the aging process. Obesity has been shown to interfere negatively in these sleep disorders, besides being able to increase obstructive sleep apnea, making the sleep repair process more difficult due to the increase in interruptions, ultimately causing poor quality sleep [9]. Lack of sleep is associated with an increased risk of falls and negative alterations in the subjective perception of vigor, fatigue, and depression [10].

In addition, depression is one of the most common mental illnesses among the older people, associated with increased premature mortality and morbidity [11]. Depression is characterized by a dysregulation of affect and mood due to dysfunction of the hypothalamic–pituitary–adrenal axis (HPA axis), which causes abnormal hormonal dynamics, a constant feature in mood disorders that could be used as a predictor of depression [12]. Trials to evaluate the dysfunction of HPA have been advantageous to obtain objective parameters for the diagnosis of mood disorders and predictions of the response to antidepressant treatment [13].

On the other hand, muscle strength plays an important role in the proper performance of the basic activities of daily life, being linked in an important way to the functional performance of older people and the feeling of achievement [14]. HGS has been used as an indicator of global strength, although there are associated parameters that significantly reduce the grip strength, such as an increase in the fat mass index, increasingly advanced age and female sex [15]. HGS is also related to nutritional status and, especially, to the Mediterranean diet, where it has been observed that older women with greater adherence to the Mediterranean diet had higher values of HGS [16]. It is also used as a predictor of functional alterations in older people [17,18].

It has been observed that HGS in a centennial population had a strong association with physical function and mood, where higher levels of HGS corresponded with a positive mood and continuity in carrying out daily activities [19]. The predictive nature of HGS has also been analyzed, showing a direct association with the functional domains of health and cognitive and social levels, and an inverse association with depressive values. HGS can be considered a causal factor of depressive symptoms [20,21].

However, the causal relationship of HGS and certain psychological aspects, such as mood, geriatric depression, and sleep, have not been sufficiently investigated, with little research having been carried out, especially in older people [20,21], this despite there being little controversy [22].

Taking into account the above information, the objective of this study was to analyze the relationship between HGS and the psychological functioning of active female older people, specifically taking into account variables such as depression, mood and sleep. The association between these factors is described.

## 2. Materials and Methods 

### 2.1. Participants

**Figure 1:**
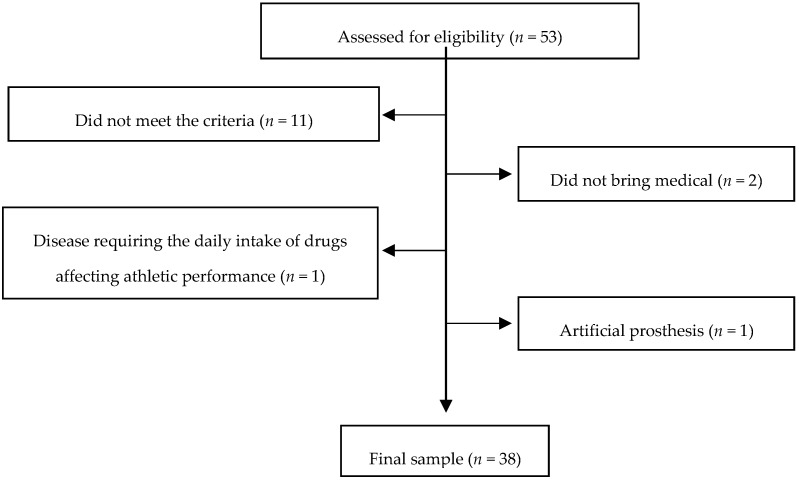
Flowchart of the participants.

In this cross-sectional study, 38 active older women (>65 years old) (age = 72.29 ± 5.21 year), from Andalusia, southern Spain, participated voluntarily. Figure 1 shows a flowchart of the study participants. The inclusion criteria were that subjects were: (I) not institutionalized; (II) active women and above the age of 65; (III) not suffering from mental and/or intellectual disorders; (IV) free of cardiovascular and neuromuscular disorders; (V) considered physically independent according to the Spanish version of the Barthel Index [23]. The exclusion criteria were: (a) participation in other training programs; (b) artificial prosthesis; (c) any disease requiring the daily intake of drugs affecting athletic performance, in order to avoid any influence on fitness measures; (d) any disease that contraindicated the exercise program; (e) any symptom that a medical professional deemed as warranting exclusion. The study was conducted in compliance with the standards of the Declaration of Helsinki (version 2013) and following the guidelines of the European Community for Good Clinical Practice (111/3976/88 of July 1990), as well as the Spanish legal framework for clinical research in humans (Royal Decree 561/1993 on clinical trials). It was approved by the Bioethics Committee of the University of Jaén (DIC. 17/3.TFM). All subjects completed an informed consent form to participate in the study.

### 2.2. Materials and Instruments

As anthropometric parameters we analyzed the height (cm), which was measured with a stadiometer (Seca 222, Hamburg, Germany). The weight (kg) was registered using a Dry scale 634 (Hamburg, Germany). The body mass index (BMI) was obtained from the equation, BMI = weight (kg)/height (m)². In addition, sociodemographic information was recorded using a self-report instrument.

The state of mind was assessed using the Profile of Mood Status (POMS) [24] in the 29-item version, adapted and validated in Spanish [25]. In this version, the dimensions that make up the test are tension, anger, vigor, fatigue and depression. The score of the scale is between 0 to 4 points for each item.

Depression was evaluated using the geriatric depression scale (GDS) [26], which consists of 15 items with a dichotomous response pattern (yes or no) that investigates the cognitive symptoms of a major depressive episode during the last fifteen days. The Spanish version was used [27]. Sleep was evaluated using the Oviedo sleep questionnaire, which is a diagnostic aid questionnaire for insomnia and hypersomnia sleep disorders. It is composed of three subscales: subjective satisfaction of sleep, insomnia and hypersomnia. In addition, it contains two items that provide information on the use of sleep aids or the presence of adverse phenomena during sleep. All the items are answered by a Likert scale, from 1 to 5, except for item 1, which is measured from 1 to 7 [28].

For the registration of the HGS, a hand-held dynamometer with an adjustable grip was used (TKK 5101 Grip D, Takey, Tokyo, Japan). The optimal grip interval was calculated using the formula suggested by a previous study [29]. Each participant performed this test twice with each hand. The participants fully extended their arm so that it formed an angle of 30° with respect to the trunk. The maximum score (in kg) for each hand was recorded, and the average score of the left and right hand was used in the subsequent analysis.

### 2.3. Procedure

All study participants were members of a functional training program during three months, from October to December, focused on retired people consisting of three training sessions per week lasting 1 h per session. The data collection was carried out in December. All the participants were informed and signed a consent form to participate in the study. The data were collected by the same researchers individually through a personal interview in which the questionnaires were filled out respecting the confidentiality of the data and clarifying the questions that arose. The tests were carried out in the same place where the participants received their usual training, by the same researchers and using the same devices to avoid inter-observer and inter-device variability. The personal interview and questionnaire completion were carried out first, followed by the HGS test and finally, the collection of anthropometric parameters. The participants did not know the objectives of the study, in order to avoid the phenomenon of social desirability.

### 2.4. Statistical Analysis

The data were analyzed using the statistical program SPSS v.19.0 for Windows (SPSS Inc, Chicago, IL, USA). The level of significance was *p* ≤ 0.05. Descriptive statistics are represented as mean and standard deviation (*SD*). Tests of normal distribution and homogeneity (Kolmogorov–Smirnov and Levene’s, respectively) were calculated on all study data before statistical analysis. A cluster analysis was performed (K-means) taking into account the performance in the HGS test, forming two groups, a first group of high HGS and another of low HGS. In addition, an analysis of covariance (ANCOVA) was performed for each psychological variable adjusting for age and BMI; moreover, effect sizes for the group differences were calculated using Partial Eta Squared. A partial Pearson correlation analysis was performed between HGS with geriatric depression, mood and sleep, adjusting for age. Finally, simple lineal regressions between HGS, vigor, depression and sleep total score were carried out.

## 3. Results

The sociodemographic variables showed that 77.8% did not complete primary studies, 42.1% were married, 94.7% did not smoke, 94.4% did not consume alcoholic beverages, 18.4% had suffered falls in the last year and finally, the perceived health (1–5, from better to worse) was 3.47 ± 0.68.

Table 1 shows the results for age, BMI, HGS, geriatric depression, mood and sleep taking into account the groups of high HGS and low HGS. The high HGS group showed better results for vigor (*p* = 0.050), depression (*p* = 0.040), insomnia (*p* = 0.032) and in the total score of the sleep scale (*p* = 0.045).

**Figure 2:**
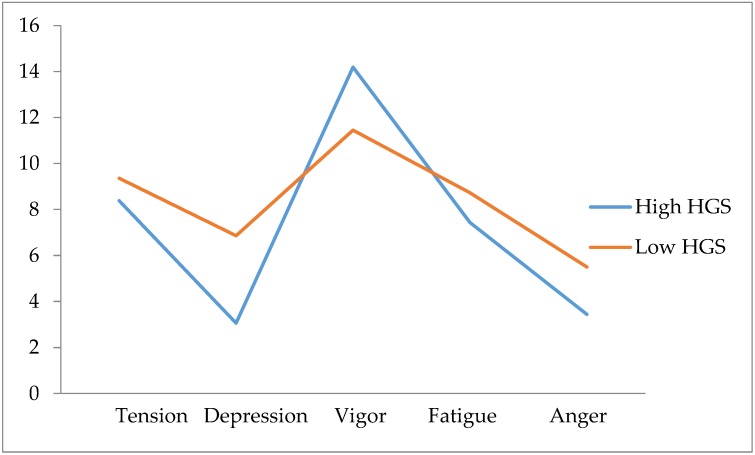
Profile of mood in high HGS and low HGS group.

**Figure 3:**
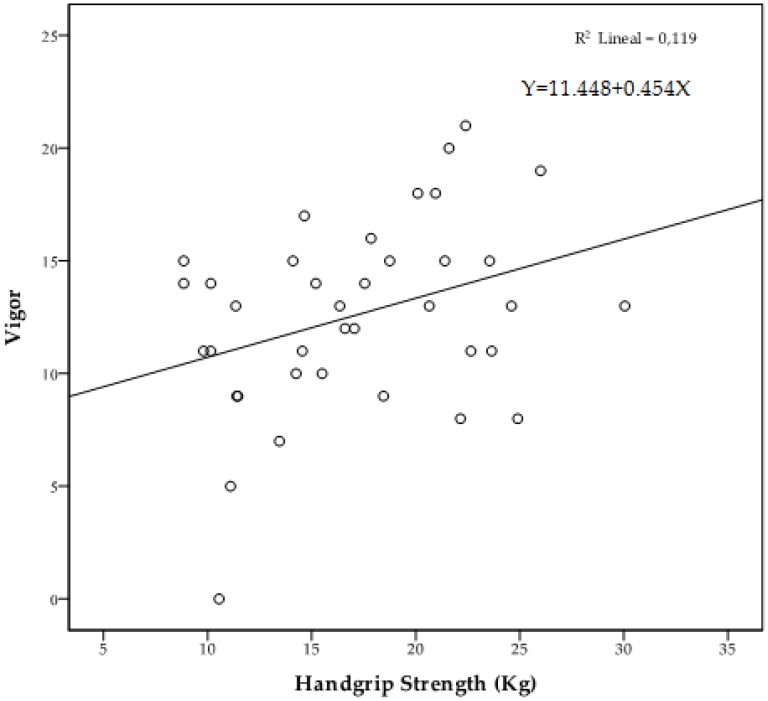
Scatter plot of HGS in relation to vigor.

**Figure 4:**
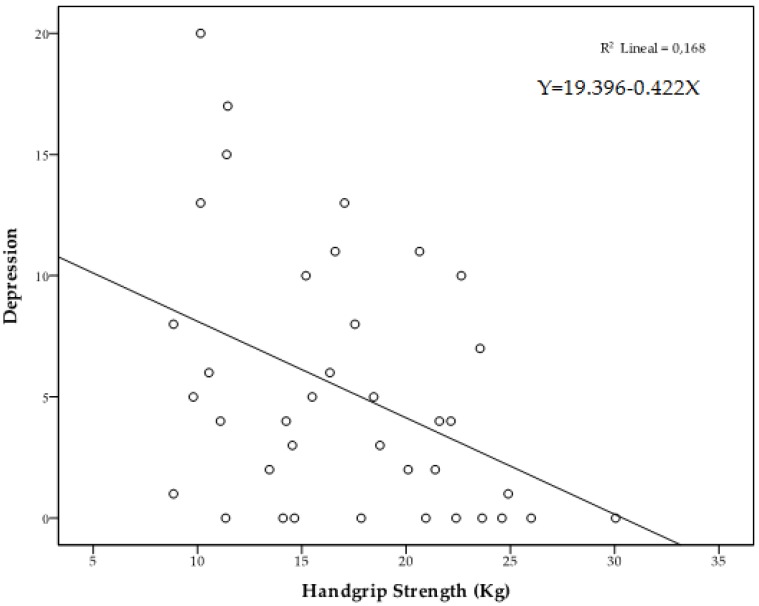
Scatter chart of HGS in relation to depression/sadness.

**Figure 5:**
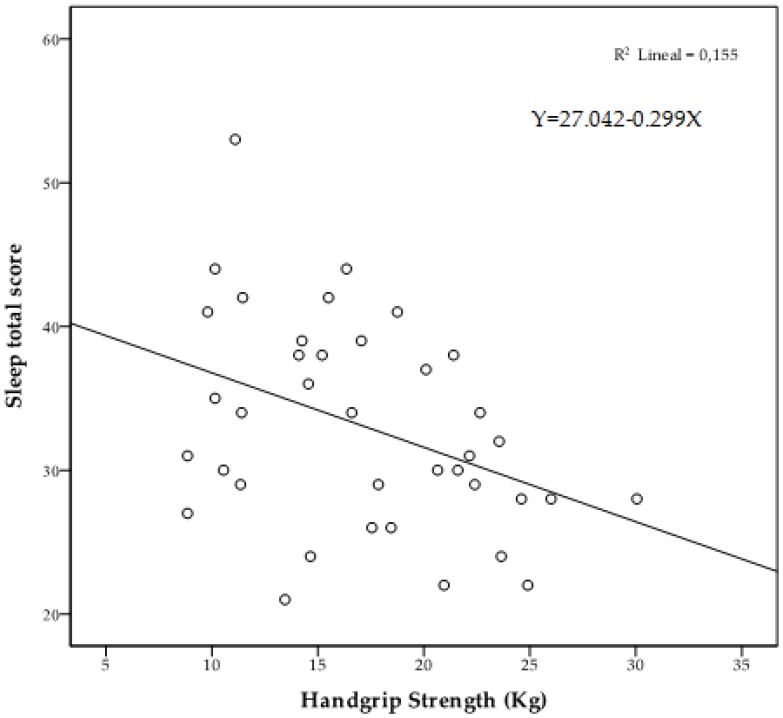
Scatter chart of HGS in relation to sleep total score.

Figure 2 shows the iceberg profile that was observed in the high HGS group. The Pearson correlation analysis adjusting for age showed significant correlations between HGS and vigor (*r* = 0.352, *p* = 0.033), depression (*r* = −0.379, *p* = 0.021) insomnia (*r* = −0.352, *p* = 0.033) and sleep total score (*r* = −0.349, *p* = 0.035). Figure 3, Figure 4 and Figure 5 represent the scatter plot for HGS, vigor, depression and sleep total score.

## 4. Discussion

The aim of this study was to analyze the relationship between HGS and the psychological functioning of older people, in particular taking into account geriatric depression, mood, and sleep. The main finding of this study indicated that HGS could be a significant predictor of mood, with a significant association with vigor, depression, and sleep quality.

To the authors’ knowledge, this is the first study that analyses the association between HGS and depression, mood, and sleep in older people. Some studies have associated HGS with functional capacity in people older than 60 years, with cognition in the general population, with depression in a population between 40 and 79 years, with perceived health in centenarians, and with psychological and social health in individuals over 85 years of age [19,20,21,22,30], with some variables from these studies being related to the present study. However, these studies did not consider the older people exclusively or the results that HGS can provide for the variables in the present study.

Franke et al. [19] analyzed the association between the perception of health status, HGS, physical activity, BMI, and positive and negative mood in older people who were centenarians. State of mind is the common variable with the present study, although for a higher age range. However, the results obtained were similar, namely that HGS is strongly associated with physical function and mood; finding that higher HGS was associated with fewer functional limitations. It may be that centenarians with a positive mood continue to do all the daily activities that they can, while a person with a negative state of mind may be more prone to quit. Taekema [20] analyzed whether HGS could predict changes in functional, psychological, and social health, finding that lower HGS was correlated with low values in functional health domains, low psychological levels such as cognition, low social levels, and high depressive values. Fukumori [21] also examined the association between HGS and mental health, focusing on depression and finding that people with lower HGS were more likely to suffer from depressive symptoms before beginning the study. The study also found an association with the development of these depressive symptoms in the future, which suggests that a decrease in HGS can be considered as a causal factor for the development of depressive symptoms. In this regard, the results of the present study related to depressive levels are in accordance with those of Taekema and Fukumori. However, Mancilla Solorza et al., [22] in their study of individuals aged between 60 and 91 years, found that there was no association between mental disorders, such as cognitive impairment, and HGS. These results might be derived from the different technique used in the HGS measurement, which in the present study was carried out with the arm completely stretched and forming an angle of 30° with respect to the trunk, as specified by a previous study that analyzed the optimal performance of this technique [29], whereas Mancilla Solorza employed a sitting technique, with the shoulder and forearm in a neutral position and with a 90° bend of the elbow [22].

In other studies, an association between low physical function (assessed by muscle strength index in the lower extremities), self-perceived functional decline, gait speed, and depressive symptoms has been identified [31,32]. As in this study, a recent study by Latorre Román et al. suggested a bidirectional association between depressive symptoms and HGS; they analyzed and found a correlation of HGS with the speed of walking [32].

As previously mentioned, several studies have showed the possible predictive character of insomnia for depression [10,33] and how people with insomnia or reduced sleep quality may have a higher risk of depression throughout their lives [34]. Scott [10] analyzed the deprivation of 30 h of sleep in six students divided into two groups, one that remained sedentary and one that performed an exercise cycle of 20 min every 2 h. In the results, no significant differences were found between the groups with respect to mood and depressive symptoms in the interval of sleep deprivation, but on both scales the levels increased as the sleep deprivation became greater, reaching their highest values in depression and fatigue at 26 h of sleep deprivation. However, with regard to vigor, the highest value was obtained at 6 h of sleep deprivation. The results of the present study show that with high HGS values, higher sleep satisfaction scores were obtained, while for low HGS values a higher score for insomnia and hypersomnia was obtained. This shows a direct association between HGS and sleep data that the present authors have not been able to contrast with previous studies because, to their knowledge, this association has not been previously studied. It is also true that sleep disorders are affected by obesity and a higher rate of BMI, since excessive weight can hinder breathing during sleep and cause obstructive sleep apnea, which causes people suffering from it to have greater sleep interruption, lower quality of sleep and daytime sleepiness [9]. However, in the present study it was observed that there were no differences between both the high and low HGS groups in terms of BMI. In addition to the BMI, it has been studied how a higher index of fat mass affects HGS showing that lower values of HGS are obtained when the fat mass index is higher [15]. In the present study, a study of the bioimpedance of the subjects was not carried out, which could be considered a limitation.

The simplicity and speed of the realization and obtaining of the results of the HGS makes it an instrument with incredible potential to identify diseases early and thus facilitate an early treatment, being able to avoid a greater severity. Other studies have revealed the predictive nature of HGS for sarcopenia [35], and mortality [36,37], and in the present study one can observe the predictive character versus depressive levels, mood and sleep, although the results must be interpreted with caution due to the study´s exploratory nature.

A very important limitation of this study is the small size of the sample, which did not allow us to extrapolate the findings of the present study. Another important limitation is that only physically active women participated in the current work. In the present study, there was no control over the nutritional intake of the participants, which could have effects on the HGS values, this must also be taken into account as another limitation. Notwithstanding these limitations, a strength of this study was the inclusion of several psychological variables in a sample of active older women.

## 5. Conclusions

In conclusion, HGS in females older than 65 years was associated with psychological functioning and sleep quality. The authors suggest further investigations on HGS to contrast the exploratory nature of this study and to verify if the monitoring of HGS could be used as a predictive indicator of psychological functioning.

## Figures and Tables

**Table 1:** Results of age, BMI, HGS, geriatric depression, mood and sleep taking into account the groups of high HGS and low HGS.

	High HGS Mean (SD) *n* = 16	Low HGS Mean (SD) *n* = 22	*p*-Valor	Partial Eta^2^
Age (years)	71.00 (5.47)	73.32 (4.89)	0.157	0.055
BMI (Kg/m^2^)	30.98 (6.37)	30.48 (5.28)	0.791	0.002
Handgrip strength (Kg)	22.61 (2.89)	13.21 (2.93)	<0.001	0.727
Geriatric depression	3.06 (2.72)	4.23 (2.54)	0.138	0.064
Vigor	14.19 (4.18)	11.45 (3.88)	0.050	0.105
Anger	3.44 (4.76)	5.50 (4.72)	0.202	0.048
Depression	3.06 (3.60)	6.86 (5.90)	0.040	0.118
Tension	8.38 (3.68)	9.36 (4.34)	0.408	0.020
Fatigue	7.44 (5.39)	8.73 (3.48)	0.197	0.048
Sleep satisfaction	3.81 (1.37)	3.82 (1.18)	0.942	<0.001
Insomnia	21.00 (4.89)	26.18 (6.58)	0.032	0.129
Hypersomnia	5.19 (1.94)	5.27 (3.04)	0.687	0.005
Sleep total score	30.00 (5.46)	35.27 (7.70)	0.045	0.113

BMI (body mass index) SD (standard deviation).
